# Baseline Soluble Anti-erythropoietin Antibody Level Is an Independent Associated Factor for Follow-Up Erythropoietin Demand in Maintenance Dialysis Patients With End-Stage Renal Disease: A Prospective Cohort Study

**DOI:** 10.3389/fmed.2020.00109

**Published:** 2020-04-07

**Authors:** Ying Zhang, Shi-Zhu Bian, Kun Yang, Yiqing Wang, Sha Tang, Weili Wang, Daihong Wang, Ling Nie, Jinghong Zhao

**Affiliations:** ^1^Department of Nephrology, Institute of Nephrology of Chongqing and Kidney Center of PLA, Xinqiao Hospital, Army Medical University (Third Medical University), Chongqing, China; ^2^Department of Cardiology, Institute of Cardiovascular Diseases of PLA, Xinqiao Hospital, Army Medical University (Third Military Medical University), Chongqing, China

**Keywords:** anti-erythropoietin antibody, predictor, erythropoietin demand, maintenance dialysis, cohort study

## Abstract

**Aims:** The aim of this study was to identify the predictive role of baseline anti-erythropoietin (anti-EPO) antibody levels in follow-up EPO demand in maintenance dialysis patients with end-stage renal disease (ESRD).

**Methods:** Baseline routine blood parameters, clinical data, dialysis-related parameters, EPO, anti-EPO antibody, and anti-EPO-receptor antibody were also measured. Differences in the abovementioned variables were compared among four intervals of the EPO demand index (EDI). Further univariate and adjusted logistic regression analyses were performed to identify the independent predictors for higher EPO demand.

**Results:** The predialysis potassium ion concentration was significantly higher in the fourth quartile (Q4) population than in the other three populations (*p* < 0.05). Furthermore, the anti-EPO antibody level showed significant differences among the four intervals (*p* = 0.006). The baseline anti-EPO antibody level was correlated with the follow-up EDI (*r*^2^ = 0.0377, *p* = 0.030). Furthermore, the follow-up EDI was significantly higher in the anti-EPO antibody-positive group (*p* = 0.02). Age (OR = 1.071, *p* = 0.005), ferritin (OR = 1.001, *p* = 0.038), potassium ion concentration before dialysis (OR = 2.781, *p* = 0.012), dialysis duration (OR = 1.025, *p* = 0.030), and anti-EPO antibody level (OR = 7.694, *p* = 0.004) were potential predictors for higher EPO demand. After adjustment, age (OR = 1.072, *p* = 0.026), potassium ion concentration before dialysis (OR = 3.425, *p* = 0.013), and EPO level (OR = 5.27, *p* = 0.007) were independent predictors for higher EDI demand.

**Conclusion:** The baseline anti-EPO antibody level combined with an older age and a higher predialysis potassium ion concentration are independent predictors for a higher follow-up EPO demand in maintenance dialysis patients with ESRD.

## Introduction

Anemia is considered the most frequent complication in patients with end-stage renal disease (ESRD), especially in the subpopulation on maintenance dialysis (1). It has also been identified as an independent risk factor/predictor for major cardiovascular events, including heart failure and atherosclerosis (2). Anemia in ESRD patients has also been demonstrated to be caused mainly by insufficient synthesis of erythropoietin (EPO) combined with erythropoietin resistance as well as a higher erythropoietin demand (3).

It has been demonstrated that up to 10% of patients in the erythropoietin-stimulating agent (ESA)-treated population have a lower erythropoietin response (1, 4, 5). Furthermore, an imbalance between the production of EPO and the demand for EPO is another pivotal reason for anemia in patients treated with ESA.

Erythropoietin demand reflects the need for erythropoietin (including endogenous and exogenous EPO) in ESRD patients in order to produce sufficient Hb to eliminate anemia (6, 7). Previous studies have shown that maintenance of dialysis patients with ESRD may be characterized by a greater erythropoietin demand or require a larger amount of ESA (1, 6). The erythropoietin demand index (EDI) is an indicator of the requirement for EPO, which is calculated as plasma erythropoietin units divided by the hemoglobin value (1).

The erythropoietin demand is a critical risk factor for cardiovascular disease; thus, it is urgent to identify the main causes for erythropoietin demand and epidemiology of increased EPO demand in maintenance dialysis patients with ESRD and its risk predictors/factors (2, 4). However, the risk factors and predictors for increased erythropoietin demand have been comprehensively investigated.

In recent decades, the anti-EPO antibody and anti-EPO receptor (anti-EPOR) antibody have been reported to be associated with EPO resistance (8–10). In addition, they may play an important role in anemia among maintenance dialysis patients combined with insufficient EPO production (6, 11). Furthermore, the roles of anti-EPO and anti-EPOR antibodies in EPO demand have not been identified. Thus, we performed the current prospective cohort study to identify the predictive role of anti-EPO and anti-EPOR antibodies in EPO demand in maintenance dialysis patients.

## Methods

### Study Design and Population

Our present research is a prospective cohort study in consecutive patients with ESRD who were undergoing maintenance dialysis at Xinqiao Hospital, Army Medical University (Third Military Medical University) from March 1, 2016, to July 1, 2019. A total of 129 consecutive patients were included in our cohort according to the inclusion and exclusion criteria. Most of the subjects were followed up within 39 months (fewer of them were followed up within 2 months). Median and quartiles of the follow-up time were 39 (39–39) months. During a median of 39 months of follow-up, nine patients were excluded or lost to follow-up.

**Figure 1:**
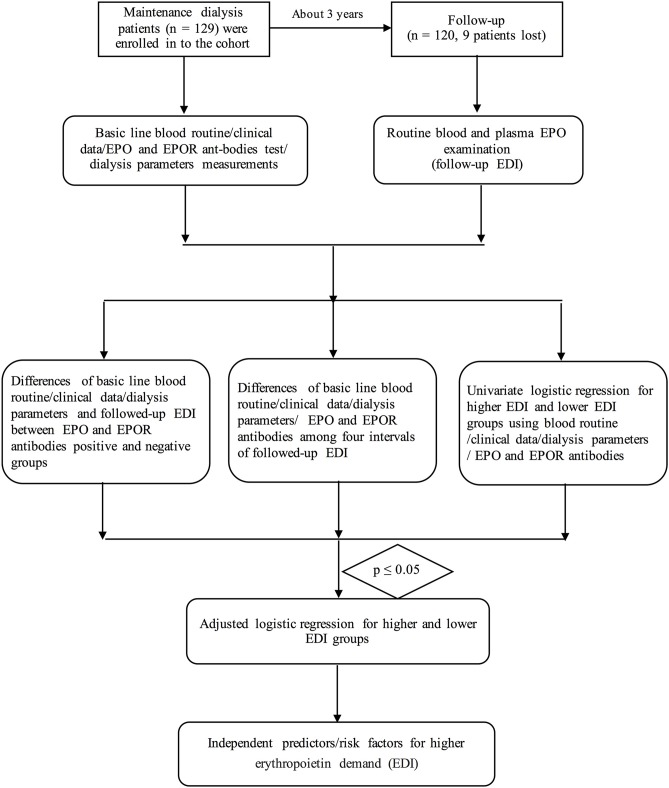
The flow chart of this study.

The inclusion criterion was ESRD patients who were undergoing maintenance dialysis. The exclusion criteria were as follows: severe hepatic dysfunction (*n* = 2), death during hospitalization (*n* = 2), advanced cancer (*n* = 1), or severe infection (1). Additionally, three patients were lost to follow-up in the cohort, as shown in Figure 1.

All patients provided written informed consent. Our present research complied with the Declaration of Helsinki with respect to human investigations and was approved by the ethics committee of Xinqiao Hospital, Army Medical University (Third Military Medical University).

#### Procedures and Clinical Data Collection

The selected maintenance dialysis patients were viewed in clinical reception by our trained physicians, Dr. Ying Zhang and Dr. Yiqing Wang, by using standardized case file records to record demographic data (age, height, and weight), lifestyle factors (smoking and alcohol consumption status), prevalent diseases (hypertension, cardiovascular diseases, and other diseases), family histories, and medication use (antihypertension drugs, anticoagulants, and phosphate binders), as well as EPO usage.

### Biomarker Variable Determination

Venous blood samples were obtained from the patients in the early morning after at least 12-h fasting at both cross sections (after entrance to the cohort and follow-up cross section) within a median of 39 months of follow-up (from 2 to 39 months).

First, we performed routine blood examination [tests of red blood cell count (RBC), mean corpuscular volume (MCV), concentration of hemoglobin (Hb), mean corpuscular hemoglobin (MCH), hematocrit (HCT), MCH concentration (MCHC), red blood cell distribution width (RDW), white blood cell count (WBC), platelet count (PLT), plateletcrit (PCT), and platelet distribution width (PDW)] by using an automated hematology corpuscle analyzer (AU400; Olympus Optical, Co., Tokyo, Japan).

Second, we measured plasma creatinine (Cr, enzyme method), UA (colorimetry), parathyroid hormone (PTH; chemiluminescent immunoassay), and ferritin (chemiluminescent immunoassay) concentrations by using Roche Diagnostics GmbH products (Abbott, i2000, USA).

Third, we also performed examinations of serum iron concentration (FERENE methods, Beckman AU5821), blood urea nitrogen concentration (BUN), potassium concentration (K^+^), and sodium concentration (Na^+^) by using indirect ion-selective electrode methods (EX-Z, JOKOH, Japan). Serum calcium concentration (Ca^2+^) was measured by using Tri-azo methods, and phosphate concentration (P) was measured with a phosphomolybdate ultraviolet method (Roche Diagnostics GmbH, USA).

Finally, EPO, anti-EPO antibody, and anti-EPOR antibody were measured using ELISA kits (Recombinant Human Erythropoietin, BioLegend and Recombinant Human R Erythropoietin, RD Systems, USA). Each sample was assayed in duplicate to measure the exact concentrations of endogenous EPO, anti-EPO antibody, and anti-EPOR antibody.

All of the biochemical variables were measured from blood specimens in the Clinical Laboratory Department, Xinqiao Hospital.

### Definition of the Variables

KT/V was calculated as ln(posturea/preurea) – 0.008 ^*^ultrafiltration time + (4 – 3.5 ^*^posturea/preurea) ^*^(ultrafiltration volume/postweight). Follow-up EPO demand was assessed by using the endogenous EPO level divided by Hb (EDI). Anti-EPO antibody positive (EPOA+) was defined as an optical density (OD) >0.5526 [the mean ± 3 standard deviations (SDs) in 55 age-matched healthy volunteers]; otherwise, it was defined as anti-EPO antibody negative (EPOA–). Similarly, EPOR antibody positive (EPOA+) was defined as an OD >0.5356 (the mean ± 3 SDs in 55 age-matched healthy volunteers); otherwise, it was defined as anti-EPOR negative (EPORA–).

### Statistical Analysis

Continuous variables (such as age and BMI) that are normally distributed were expressed as the means ± SD. The differences between two groups or among four EDI intervals of these variables were compared using independent Student's *t*-test or one-way ANOVA [comparisons between two intervals were made using least significant difference (LSD) methods]. The Wilcoxon rank sum test was used to compare the non-normally distributed variables (comparisons among four intervals were performed by using one-way ANOVA after transformation to normality). Furthermore, the chi-square test or Fisher's exact test (if there were less than five cases) was employed to assess dichotomous variables. Univariate logistic regression analyses were performed to primarily screen the risk factors for EPO demand. Variables with *p* < 0.05 in comparisons among different EDI groups or in the univariate logistic regression were included in multivariate (adjusted) logistic regression analyses to identify independent associated factors of higher EPO demand. The regressions were performed using ordinal logistic regression analyses, and we have just listed the Q4 EDI vs. other intervals (Q1–Q3) owing to too many data. Furthermore, the generalized linear model's analyses have also been performed to determine the OR and its 95% CI. All of the statistical analyses were performed with SPSS 22.0 for MAC statistical software (California, USA).

## Results

At the follow-up cross section, 120 patients were included in the statistical analysis. The basic characteristics of the patients are listed in Table 1. The mean age of the subjects was 48.17 ± 13.55 years, and the BMI was 21.97 ± 3.24 kg/m^2^. A total of 77 patients were diagnosed with anemia (64.2%). Hypertension status and drug use are also shown in Table 1. The follow-up endogenous EPO was 18.05 (10.70–18.88) g/dl in these maintenance dialysis patients.

**Table 1:** Characteristics of the patients.

**Basic information of the patient (*n* = 120)**	
Age (years)	48.17 ± 13.55
BMI (kg/m^2^)	21.97 ± 3.24
Gender (female)	48 (40%)
Smoker (cases and %)	7 (5.8%)
Alcohol use (cases and %)	5 (3.3%)
Dialysis duration (month)	34.50 (22–65)
Anemia (cases and %)	77 (64.2%)
Hypertension (cases and %)	38 (31.7%)
ACEI/ARB (cases and %)	18 (15.0%)
CCB (cases and %)	30 (25%)
β receptor blocker (cases and %)	19 (15.8%)

*ACEI, angiotensin-converting enzyme inhibitor; ARB, angiotensin receptor blocker; CCB, calcium channel blockers*.

### Differences in Baseline Parameters Among the Four Intervals of Follow-Up Erythropoietin Demand Index

The EDI was divided into four quartile intervals, and we compared the differences among various groups. The patients in the Q4 and Q3 groups were significantly older than those in the Q2 and Q1 groups (*p*-values were <0.05; Table 2).

**Table 2:** Differences of basic line routine blood, clinical data, dialysis parameters, EPO, and EPOR antibodies among four intervals of follow-up EDI.

	**Overall** **(*n* = 120)**	**Q1** **(*n* = 30)**	**Q2** **(*n* = 30)**	**Q3** **(*n* = 30)**	**Q4** **(*n* = 30)**	***p*-value** **(among four intervals)**
**DEMOGRAPHICS**						
Age (years)	48.17 ± 13.55	43.01 ± 12.52	46.00 ± 14.10	51.33 ± 15.03^*^	52.34 ± 10.53^#^	0.020
BMI (kg/m^2^)	21.97 ± 3.24	21.74 ± 3.16	22.51 ± 3.10	21.99 ± 3.62	21.63 ± 3.15	0.730
**BASELINE DATA IN THE INITIAL OF THE STUDY**						
**Blood routine examination parameters**						
RBC (10^12^/L)	3.45 ± 0.61	3.59 ± 0.64	3.48 ± 0.51	3.40 ± 0.64	3.34 ± 0.63	0.407
Hb (g/dl)	10.40 (9.02–11.58)	10.95 (9.82–12.02)	10.45 (9.12–11.18)	10.20 (9.08–11.42)	10.30 (8.38–11.6.5)^*^^#^^*Δ*^	0.012
Hct (L/L)	33.52 ± 5.38	34.61 ± 6.13	33.31 ± 5.10	33.31 ± 4.97	32.85 ± 5.36	0.623
MCV (fl)	98.10 (93.60–102.27)	96.65 (93.025–99.05)	96.60 (92.75–203.77)	98.95 (92.70–102.15)	99.25 (95.00–103.27)	0.520
MCH (pg)	30.95 (29.15–32.27)	30.50 (29.25–31.20)	30.95 (28.95–32.35)	31.30 (29.62–32.67)	31.05 (29.70–32.95)	0.542
MCHC (g/dl)	313.00 (307.25–321.75)	313.50 (309.75–321.25)	311.00 (307.00–321.50)	315.00 (308.75–322.25)	311.50 (303.00–320.50)	0.227
RDW-CV (%)	14.43 ± 1.39	14.62 ± 1.32	14.41 ± 1.34	14.12 ± 1.26	14.56 ± 1.64	0.512
WBC (10^9^/L)	6.26 ± 1.66	6.36 ± 1.82	6.20 ± 1.45	6.20 ± 1.78	6.30 ± 1.65	0.997
PCT (%)	0.155 (0.122–0.190)	0.160 (0.120–0.190)	0.165 (0.125–0.192)	0.140 (0.117–0.210)	0.160 (0.130–0.190)	0.970
PLT (10^9^/L)	177.50 (134.00–215.75)	163.00 (135.75–219.25)	185.50 (132.50–212.50)	156.50 (121.75–221.75)	185.50 (131.00–214.50)	0.988
MPV (fl)	9.14 ± 1.35	9.20 ± 1.38	9.12 ± 1.42	9.08 ± 1.31	9.12 ± 1.37	0.988
PDW (%)	16.30 (16.00–16.60)	16.30 (15.90–16.50)	16.30 (16.00–16.50)	16,45 (16.07–16.77)	16.25 (16.00–16.52)	0.865
**Clinical data**						
Serum iron (μmol/L)	13.37 (10.00–15.30)	13.45 (10.77–16.25)	11.25 (8.67–13.45)	12.20 (10.07–17.45)	13.45 (10.25–17.82)	0.113
Ferritin (μg/L)	383.28 (161.19–656.76)	344.80 (91.56–560.51)	298.30 (162.85–524.19)	414.53 (213.26–631.64)	524.17 (265.50–838.53)	0.069
PTH (pg/ml)	499.5 (237.75–800.5)	433.00 (243.75–846.5)	414.5 (228.75–849.75)	641.73 (247.5–1,065.25)	431.5 (229.25–671.75)	0.509
**Dialysis parameters**						
Dialysis duration (months)	34.5 (22.0–65.0)	26.0 (16.25–46.5)	37.5 (23.75–52.75)	34.0 (21.75–66.25)	47.5 (22.75–75.0)^*^	0.179
Ultrafiltration volume (L)	2.00 (0.00–2.80)	2.55 (0.00–2.92)	2.40 (0.00–3.00)	1.70 (0.00–2.72)	2.00 (0.00–2.800)	0.640
**Predialysis**						
Urea (μmol/L)	26.42 ± 5.88	26.85 ± 5.19	26.84 ± 6.70	25.45 ± 5.68	26.52 ± 6.03	0.772
Cr (μmol/L)	1,030.20 ± 248.52	1,091.82 ± 230.90	1,086.84 ± 298.61	967.02 ± 219.54	975.12 ± 219.66	0.076
Uric acid (μmol/L)	506.47 ± 90.20	519.92 ± 99.62	519.67 ± 101.75	481.23 ± 62.02	505.06 ± 90.74	0.302
Calcium ion (mmol/L)	2.24 (2.07–2.35)	2.25 (2.07–2.35)	2.25 (2.11–2.35)	2.24 (2.07–2.39)	2.15 (2.00–2.33)	0.496
Serum phosphate (mmol/L)	2.21 (1.74–2.63)	2.42 (1.98–2.70)	2.08 (1.74–2.55)	1.94 (1.66–2.50)	2.31 (1.73–2.65)	0.240
Potassium ion (mmol/L)	5.15 ± 0.77	5.00 ± 0.68	5.01 ± 0.76	5.08 ± 0.79	5.52 ± 0.76^*^^#^	0.024
Serum sodium (mmol/L)	137.86 ± 3.00	137.27 ± 2.69	138.10 ± 2.34	138.81 ± 3.20	137.25 ± 3.50	0.132
TCO_2_ (mmol/L)	20.41 ± 3.07	20.55 ± 3.32	19.78 ± 2.62	20.99 ± 3.72	20.33 ± 2.51	0.500
**Postdialysis**						
Urea (mmol/L)	9.09 ± 3.04	9.26 ± 2.64	9.64 ± 3.74	8.53 ± 2.69	8.89 ± 3.00	0.853
Cr (μmol/L)	388.20 ± 140.76	402.51 ± 137.07	420.17 ± 157.01	371.64 ± 130.28	358.47 ± 135.73	0.309
Uric acid (μmol/L)	141.99 ± 40.82	138.97 ± 44.22	145.83 ± 42.70	138.39 ± 41.98	144.75 ± 35.37	0.853
Calcium ion (mmol/L)	2.38 (2.26–2.49)	2.40 (2.27–2.45)	2.40 (2.28–2.50)	2.32 (2.16–2.45)	2.35 (2.17–2.51)	0.509
Serum phosphate (mmol/L)	0.83 (0.65–1.01)	0.84 (0.71–0.97)	0.82 (0.60–0.98)	0.82 (0.62–1.01)	0.91 (0.66–1.11)	0.877
Potassium ion (mmol/L)	3.51 ± 0.38	3.49 ± 0.37	3.43 ± 0.35	3.51 ± 0.37	3.61 ± 0.42	0.310
Serum sodium (mmol/L)	138.70 ± 2.56	138.88 ± 2.55	138.83 ± 2.16	139.07 ± 2.57	137.99 ± 2.90	0.369
TCO_2_ (μmol/L)	26.38 ± 3.37	26.34 ± 3.89	26.06 ± 2.78	27.25 ± 3.44	25.87 ± 3.29	0.403
Weight (kg)	59.06 ± 10.46	58.64 ± 10.98	62.15 ± 11.43	38.38 ± 9.33	57.05 ± 9.83	0.275
KT/V	1.22 (1.08–1.45)	1.20 (1.04–1.49)	1.24 (1.05–1.45)	1.21 (1.08–1.43)	1.24 (1.14–1.45)	0.879
**EPO and EPOR antibodies**						
EPOA OD	0.428 (0.314–0.542)	0.357 (0.266–0.447)	0.426 (0.316–0.574)^*^	0.468 (0.367–0.546)^*^	0.456 (0.382–0.616)^*^	0.006
EPORA OD	0.359 (0.226–0.562)	0.413 (0.336–0.577)	0.305 (0.208–0.445)	0.413 (0.187–0.613)	0.421 (0.289–0.578)	0.387

*Cr, creatinine; EDI, erythropoietin demand index; EPO, erythropoietin, mIU/ml; EPOA, EPO antibody; EPOA+, EPO antibody positive; EPORA, EPOR antibody; EPORA+, EPOR antibody positive; Hb, hemoglobin; Hct, hematocrit; MCH, mean corpuscular hemoglobin; MCHC, mean corpuscular hemoglobin concentration; MCV, mean corpuscular volume; OD, optical density; PCT, plateletcrit; PDW, platelet distribution width; PLT, platelet; PTH, parathyroid hormone; RBC, red blood cell; RDW, red blood cell distribution width; WBC, white blood cell*.

* *p ≤ 0.05 compared with Q1*.

# *p ≤ 0.05 compared with Q2*.

Δ *p ≤ 0.05 compared with Q3*.

In the routine blood tests, only Hb showed significant differences among different EDI intervals. The Hb concentration was significantly higher in the Q1 EDI group at 10.95 (9.82–12.02). Other routine blood test parameters, including HCT, MCV, MCH, MCHC, RDW-CV, WBC, PCT, PLT, mean platelet volume (MPV), and PDW, showed no differences among the various intervals of EDI.

The serum iron, ferritin, and PTH levels were similar in the groups (all *p*-values were >0.05).

In the dialysis parameters, the dialysis duration showed no differences among the four intervals (*p* = 0.179). Predialysis, the potassium ion concentration was significantly higher in the Q4 population than in the other three populations (all *p*-values were <0.05). However, other predialysis parameters, including the concentrations of urea, Cr, uric acid, calcium ion, serum phosphate, and serum sodium, as well as TCO_2_, showed no differences among these populations (all of the *p*-values were >0.05). Furthermore, the postdialysis-related variables were all similar among various populations, including concentrations of urea, Cr, uric acid, calcium ion, serum phosphate, potassium ion, and serum sodium, as well as TCO_2_.

Unfortunately, we found no difference in either ultrafiltration volume or KT/V value (*p* > 0.05). Focusing on the target level of anti-EPO and anti-EPOR antibodies, only anti-EPO antibody showed significant differences among the four intervals (*p* = 0.006); that is, the levels of anti-EPO antibody were significantly higher in the Q2, Q3, and Q4 populations than in the Q1 subgroup (all *p*-values were <0.05). However, the anti-EPO antibody levels were similar among the three subgroups (Q2, Q3, and Q4, Table 2).

### Differences in Baseline Parameters Between Erythropoietin and Erythropoietin Receptor Antibodies Positive and Negative Groups

We further analyzed the differences in baseline parameters between EPO/EPOR antibody-positive and EPO/EPOR antibody-negative groups. First, age and BMI showed no difference between the EPOA+ and EPOA– groups or between the EPORA+ and EPORA– groups.

In the routine blood examination parameters, we found that only RDW was significantly higher in the EPOA+ group than in the EPOA– group (14.87 ± 1.57% vs. 14.28 ± 1.31%, *p* = 0.046). Only one variable (MPV) was significantly higher in the EPORA+ group than in the EPORA– group (9.65 ± 1.70 vs. 8.94 ± 1.14 fl, *p* = 0.010). However, none of the other routine blood examination parameters showed any differences between the EPORA+ and EPORA– individuals (*p* > 0.05).

Moreover, neither clinical data (serum iron, ferritin, and PTH) nor dialysis-related parameters showed any differences between the EPOA+ and EPORA+ populations.

Regarding the EPORA groups, we found that ferritin and TCO_2_ (both predialysis and postdialysis) showed differences between the EPORA+ and EPORA– populations. Other clinical data and dialysis-related parameters were similar in the two groups (Table 3).

**Table 3:** Differences of basic line blood routine, clinical data, and dialysis parameters between EPO and EPOR antibody positive and negative groups.

	**EPOA – group** **(*n* = 91)**	**EPOA + group** **(*n* = 29)**	***P***	**EPORA – group** **(*n* = 87)**	**EPORA + group** **(*n* = 33)**	***P***
**DEMOGRAPHICS**						
Age (years)	47.69 ± 12.90	49.67 ± 15.55	0.494	46.95 ± 12.81	51.07 ± 15.06	0.109
BMI (kg/m^2^)	21.65 ± 3.05	22.96 ± 3.66	0.059	21.74 ± 3.12	22.56 ± 3.52	0.220
**BASELINE DATA IN THE INITIAL OF THE STUDY**						
**Routine blood examination parameters**						
RBC (10^12^/L)	3.44 ± 0.58	3.49 ± 0.69	0.720	3.51 ± 0.61	3.30 ± 0.59	0.099
Hb (g/dl)	104.00 (92.00–116.00)	100.00 (87.00–114.00)	0.306	104.00 (93.00–117.00)	103.00 (86.00–115.00)	0.389
Hct (L/L)	33.58 ± 5.36	33.31 ± 5.52	0.813	34.04 ± 5.31	32.14 ± 5.39	0.085
MCV (fl)	98.60 (93.60–102.90)	95.70 (93.00–102.10)	0.445	98.30 (94.00–102.20)	97.90 (92.55–102.45)	0.624
MCH (pg)	31.00 (29.40–32.30)	30.50 (28.60–31.85)	0.343	31.00 (29.10–32.10)	30.50 (29.25–32.85)	0.911
MCHC (g/dl)	314.00 (308.00–322.00)	311.00 (304.50–321.00)	0.447	313.00 (308.00–320.00)	316.00 (305.50–323.50)	0.379
RDW-CV (%)	14.28 ± 1.31	14.87 ± 1.57	0.046^*^	14.32 ± 1.33	14.70 ± 1.53	0.182
WBC (10^9^/L)	6.32 ± 1.65	6.09 ± 1.70	0.525	6.28 ± 1.73	6.23 ± 1.50	0.887
PCT (%)	0.160 (0.130–0.200)	0.150 (0.105–0.180)	0.218	0.150 (0.130–0.190)	0.160 (0.110–0.205)	1.000
PLT (10^9^/L)	178.00 (135.00–223.00)	176.73 (122.50–195.00)	0.186	177.00 (136.00–216.00)	179.00 (114.50–204.50)	0.389
MPV (fl)	9.12 ± 1.36	9.19 ± 1.34	0.811	8.94 ± 1.14	9.65 ± 1.70	0.010
PDW (%)	16.30 (16.00–16.50)	16.40 (16.10–16.70)	0.286	16.30 (16.00–16.60)	16.30 (15.65–16.65)	0.699
**Clinical data**						
Serum iron (μmol/L)	13.30 (10.00–15.80)	13.45 (9.65–13.70)	0.773	10.10 (13.45–15.80)	12.50 (9.50–14.25)	0.530
Ferritin (μg/L)	377.70 (151.26–618.55)	483.24 (170.46–765.79)	0.405	344.85 (155.85–524.19)	524.19 (182.59–1,183.17)	0.025^*^
PTH (pg/ml)	431.00 (237.00–772.00)	536.00 (238.00–981.00)	0.654	406.00 (233.00–710.00)	641.73 (363.50–1,028.50)	0.068
**Dialysis parameters**						
Dialysis duration (month)	35.0 (22.0–59.0)	34.00 (22.00–70.50)	0.832	34.00 (22.00–59.00)	39.00 (22.00–71.50)	0.352
Ultrafiltration volume (L)	2.00 (0.00–2.80)	2.40 (0.00–2.85)	0.744	2.20 (0.00–2.80)	1.00 (0.00–2.85)	0.223
**Predialysis**						
Urea (μmol/L)	26.02 ± 5.74	27.65 ± 6.24	0.196	26.43 ± 5.68	26.36 ± 6.46	0.950
Cr (μmol/L)	1,023.62 ± 241.80	1,050.86 ± 272.01	0.609	1,038.00 ± 243.08	1,009.64 ± 265.12	0.579
Uric acid (μmol/L)	506.80 ± 94.92	505.42 ± 74.92	0.943	507.83 ± 87.80	502.89 ± 97.55	0.790
Calcium ion (mmol/L)	2.24 (2.07–2.35)	2.22 (2.08–2.31)	0.773	2.22 (2.08–2.35)	2.25 (2.05–2.38)	0.962
Serum phosphate (mmol/L)	2.23 (1.74–2.64)	2.20 (1.74–2.61)	0.806	2.20 (1.74–2.64)	2.35 (1.74–2.63)	0.791
Potassium ion (mmol/L)	5.17 ± 0.80	5.08 ± 0.69	0.546	5.21 ± 0.80	4.99 ± 0.67	0.162
Serum sodium (mmol/L)	137.88 ± 3.06	137.78 ± 2.84	0.881	137.80 ± 2.87	138.00 ± 3.36	0.744
TCO_2_ (mmol/L)	20.25 ± 3.27	20.90 ± 2.34	0.327	19.95 ± 2.91	21.63 ± 3.20	0.007^**^
**Postdialysis**						
Urea (mmol/L)	8.90 ± 2.92	9.65 ± 3.39	0.254	9.06 ± 3.04	9.15 ± 3.10	0.889
Cr (μmol/L)	385.12 ± 138.25	397.85 ± 150.48	0.673	388.50 ± 142.43	387.39 ± 138.42	0.969
Uric acid (μmol/L)	140.39 ± 42.93	146.98 ± 33.51	0.452	139.20 ± 37.67	149.32 ± 48.03	0.227
Calcium ion (mmol/L)	2.38 (2.26–2.46)	2.37 (2.26–2.56)	0.734	2.39 (2.28–2.50)	2.35 (2.17–2.47)	0.448
Serum phosphate (mmol/L)	0.830 (0.650–0.980)	0.847 (0.705–1.080)	0.334	0.830 (0.660–0.980)	0.920 (0.560–1.045)	0.881
Potassium ion (mmol/L)	3.52 ± 0.39	3.48 ± 0.35	0.609	3.54 ± 0.39	3.43 ± 0.34	0.188
Serum sodium (mmol/L)	138.70 ± 2.67	138.69 ± 2.22	0.990	138.72 ± 2.63	138.63 ± 2.40	0.863
TCO_2_ (μmol/L)	26.21 ± 3.48	26.91 ± 3.01	0.334	25.99 ± 3.22	27.42 ± 3.60	0.037^*^
Weight (kg)	58.57 ± 10.09	60.57 ± 11.61	0.372	58.81 ± 10.35	59.69 ± 10.89	0.683
KT/V	1.22 (1.09–1.45)	1.21 (1.01–1.45)	0.963	1.28 (1.11–1.46)	1.15 (1.04–1.37)	0.090

*Cr, creatinine; EPO, erythropoietin, mIU/ml; EPOA, EPO antibody; EPOA+, EPO antibody positive; EPORA, EPOR antibody; EPORA+, EPOR antibody positive; Hb, hemoglobin; Hct, hematocrit; MCH, mean corpuscular hemoglobin; MCHC, mean corpuscular hemoglobin concentration; MCV, mean corpuscular volume; OD, optical density; PCT, plateletcrit; PDW, platelet distribution width; PLT, platelet; PTH, parathyroid hormone; RBC, red blood cell; RDW, red blood cell distribution width; WBC, white blood cell*.

* *p ≤ 0.05 between two groups*.

** *p ≤ 0.01 between two groups*.

### Associations Between Erythropoietin Demand Index and Erythropoietin/Erythropoietin Receptor Antibodies

**Figure 2:**
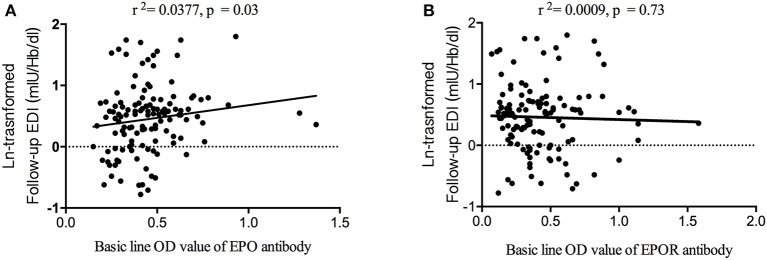
Correlation between follow-up EDI and baseline anti-EPO and anti-EPOR antibodies. **(A)** The baseline anti-EPO antibody level was correlated with follow-up EDI. **(B)** The baseline anti-EPOR antibody level was not correlated with follow-up EDI. EDI, erythropoietin demand index; EPO, erythropoietin; EPOR, erythropoietin receptor.

**Figure 3:**
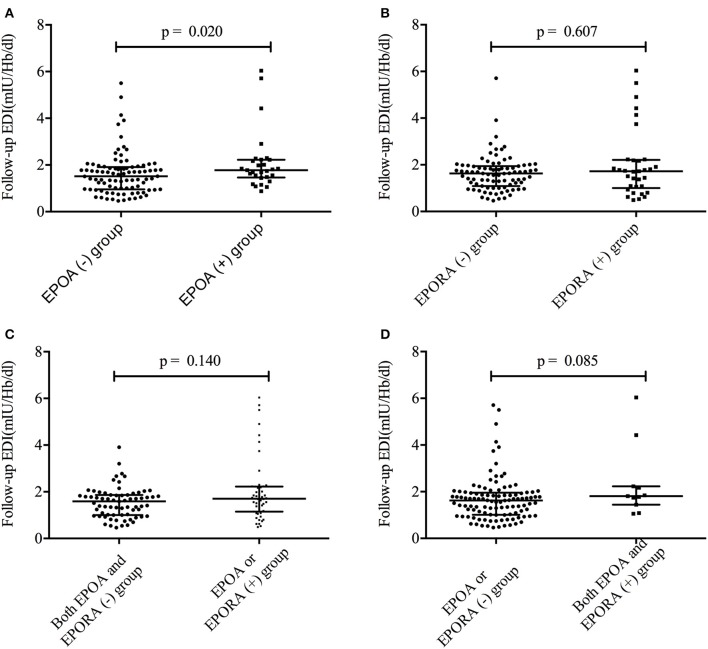
The follow-up EDI was different between the different groups. **(A)** The follow-up EDI was different between the EPOA+ and EPOA– groups. **(B)** The follow-up EDI was not different between the EPORA+ and EPORA– groups. **(C)** Follow-up EDI showed no difference between the EPOA/EPORA+ group and the EPOA– (plus EPORA–) group. **(D)** Follow-up EDI showed no difference between the EPOA+ (plus EPORA+) group and the EPOA–/EPORA– group. EDI, erythropoietin demand index; EPOA–, EPOA negative; EPOA, erythropoietin antibody; EPOA+, EPOA positive.

To identify the associations between EDI and anti-EPO/anti-EPOR antibody levels, we further compared the EDI between the EPOA + and EPOA– groups (as well as between the EPORA+ and EPORA– groups). As shown in Figure 2, the baseline EPO antibody level is correlated with follow-up EDI (*r*^2^ = 0.0377, *p* = 0.03), whereas the baseline anti-EPOR antibody level was not correlated with EDI (*r*^2^ = 0.0009, *p* = 0.73). Furthermore, in Figure 3, we found that the follow-up EDI was significantly higher in the EPOA+ group than in the EPOA– group (*p* = 0.02, Figure 3A). However, there were no significant differences between the EPORA+ and EPORA– groups. In addition, follow-up EDI was similar in the EPOA+ combined with EPORA+ group and EPOA– combined EPORA– group (Figures 3B–D). The correlations between the follow-up EDI and other parameters are shown in Table S1.

### Univariate and Adjusted Regressions for the Higher Erythropoietin Demand Index Group

To identify the predictive roles of EPO and other potential factors, we performed univariate logistic regression for each parameter between the higher and lower erythropoietin demand (Q4 of EDI vs. Q1 of EDI). Other analyses between Q3 EDI and Q1 EDI are shown in the Appendix.

In the univariate logistic regression analyses, we found that age (OR = 1.071, 95% CI 95% CI: 1.021–1.123, *p* = 0.005), ferritin (OR = 1.001, 95% CI: 1.000–1.003, *p* = 0.038), potassium ion concentration before dialysis (OR = 2.781, 95% CI: 1.255–6.162, *p* = 0.012), dialysis duration (OR = 1.025, 95% CI: 1.002–1.048, *p* = 0.030), and EPOA OD value (OR = 7.694, 95% CI: 2.109–67.277, *p* = 0.004) were potential associated factors for higher erythropoietin demand (Table 4).

**Table 4:** Univariate regressions for higher EDI (Q4).

**Variables**	**β**	***P***	**OR**	**95% CI**	
				**Lower border**	**Upper border**
**DEMOGRAPHICS**					
Age (years)	0.068	0.005^**^	1.071	1.021	1.123
BMI (kg/m^2^)	−0.011	0.899	0.990	0.841	1.165
**BASELINE DATA IN THE INITIAL OF THE STUDY**					
**Blood routine examination parameters**					
RBC (10^12^/L)	−0.642	0.129	0.526	0.230	1.205
Hb (g/dl)	−0.023	0.146	0.977	0.948	1.008
Hct (L/L)	−0.055	0.239	0.947	0.864	1.037
MCV (fl)	0.057	0.156	1.058	0.979	1.145
MCH (pg)	0.084	0.409	1.087	0.891	1.326
MCHC (g/dl)	−0.023	0.316	0.977	0.933	1.023
RDW (%)	0.025	0.902	1.026	0.686	1.535
WBC (10^9^/L)	−0.020	0.893	0.980	0.730	1.317
PCT (%)	−0.587	0.916	−0.556	0.000	32,573.031
PLT (10^9^/L)	−0.000	1.000	1.000	0.991	1.009
MPV (fl)	−0.042	0.825	0.957	0.659	1.394
PDW (%)	0.025	0.902	1.026	0.686	1.535
**Clinical data**					
Serum iron (μmol/L)	0.048	0.302	1.049	0.958	1.149
Ferritin (μg/L)	0.001	0.038^*^	1.001	1.000	1.003
PTH (pg/ml)	−0.001	0.137	0.999	0.998	1.000
**Dialysis parameters**					
Dialysis duration (month)	0.025	0.030^*^	1.025	1.002	1.048
Ultrafiltration volume (L)	−0.071	0.703	0.931	0.646	1.342
**Predialysis**
Urea (μmol/L)	−0.011	0.816	0.989	0.903	1.084
Cr (μmol/L)	−0.002	0.055	0.998	0.995	1.000
Uric acid (μmol/L)	−0.002	0.541	0.998	0.993	1.004
Calcium ion (mmol/L)	−1.197	0.272	0.302	0.036	2.553
Serum phosphate (mmol/L)	0.137	0.712	1.147	0.554	2.376
Potassium ion (mmol/L)	1.023	0.012^*^	2.781	1.255	6.162
Serum sodium (mmol/L)	−0.001	0.987	0.999	0.847	1.178
TCO_2_ (mmol/L)	−0.027	0.765	0.974	0.817	1.160
**Postdialysis**					
Urea (mmol/L)	−0.048	0.606	0.953	0.793	1.144
Cr (μmol/L)	−0.002	0.215	0.997	0.993	1.001
Uric acid (μmol/L)	0.004	0.572	1.004	0.991	1.017
Calcium ion (mmol/L)	−1.142	0.394	0.319	0.023	4.407
Serum phosphate (mmol/L)	0.569	0.587	1.767	0.227	13.751
Potassium ion (mmol/L)	0.790	0.237	2.204	0.594	8.175
Serum sodium (mmol/L)	−0.123	0.210	0.884	0.729	1.072
TCO_2_ (μmol/L)	−0.038	0.608	0.963	0.834	1.112
Weight (kg)	−0.015	0.549	0.985	0.937	1.035
KT/V	0.333	0.707	1.396	0.246	7.931
**EPO and EPOR antibodies**					
EPORA OD	0.098	0.937	1.103	0.097	12.596
EPOA OD	2.126	0.004^**^	7.694	2.109	67.277

*The regressions were performed using the Q1 interval as the reference, with Q4 as the higher EDI. The regressions between Q3 and Q1 were appended in the Appendix*.

*Cr, creatinine; EDI, erythropoietin demand index; EPO, erythropoietin, mIU/ml; EPOA, EPO antibody; EPOA+, EPO antibody positive; EPORA, EPOR antibody; EPORA+, EPOR antibody positive; Hb, hemoglobin; Hct, hematocrit; MCH, mean corpuscular hemoglobin; MCHC, mean corpuscular hemoglobin concentration; MCV, mean corpuscular volume; OD, optical density; PCT, plateletcrit; PDW, platelet distribution width; PLT, platelet; PTH, parathyroid hormone; RBC, red blood cell; RDW, red blood cell distribution width; WBC, white blood cell*.

* *p ≤ 0.05*.

** *p ≤ 0.01*.

To identify the independent associated factors of higher erythropoietin demand, variables that showed differences among various intervals of EDI or that had a *p*-value of <0.05 in univariate logistic regression analyses were included in the adjusted logistic regression. Ultimately, age (OR = 1.072, 95% CI: 1.008–1.140, *p* = 0.026), potassium ion concentration before dialysis (OR = 3.425, 95% CI: 1.297–9.040, *p* = 0.013), and EPOA OD value (OR = 5.27, 95% CI: 2.577–6.733, *p* = 0.007) were found to be independent associated factors of higher erythropoietin demand (Table 5).

**Table 5:** Adjusted regressions.

**Variables**	**β**	***P***	**OR**	**95% CI**	
				**Lower border**	**Upper border**
Age	0.070	0.026^*^	1.072	1.008	1.140
Predialysis Potassium ion	1.231	0.013^*^	3.425	1.297	9.040
EPOA OD	1.990	0.007^**^	5.27	2.577	6.733

*The regressions were performed using the Q1 interval as the reference, with Q4 as the higher EDI. The regressions between Q3 and Q1 were appended in the Appendix*.

*EDI, erythropoietin demand index; EPOA, erythropoietin antibody; OD, optical density*.

* *p ≤ 0.05*.

** *p ≤ 0.01*.

The ordinal logistic regression analyses have found that the baseline EPO antibody level (OR = 5.328, 95% CI: 1.072–8.735, *p* = 0.045), ferritin (OR = 6.746, 95% CI: 1.028–9.976, *p* = 0.044), and PTH (OR= 0.064, 95% CI: 0.009–0.148, *p* = 0.035) were independently associated with higher EDI (Tables S4, S5).

## Discussion

In the present cohort study lasting a median of 39 months, we found differences in age, baseline Hb, baseline predialysis potassium ion concentration, and baseline anti-EPO antibody level among four follow-up EDI intervals. Furthermore, EDI was significantly higher in the EPOA+ group than in the EPOA– group, and it was associated with the anti-EPO antibody level. Further analyses indicated that age, potassium ion concentration before dialysis, and anti-EPO antibody OD level were independent associated factors of higher EPO demand or higher EDI.

As mentioned in the *Introduction*, anemia is one of the most frequent complications accompanying ESRD, especially in the maintenance dialysis patient subpopulation (1, 7, 11, 12). In the present study, more than half of the maintenance dialysis patients had various severities of anemia. It is known that patients with ESRD produce insufficient EPO due to kidney dysfunction to satisfy the higher need for EPO resulting in anemia. It is also the most important reason for use of ESA.

Age has been considered a non-modifiable risk factor for various diseases, including anemia and renal dysfunction (13, 14). Without exception, we found that older patients with ESRD who underwent maintenance dialysis were characterized by a higher EDI, indicating a greater requirement for EPO. We also found that age may be the potential risk factor for higher EPO demand in the primary screening univariate regression analysis. Final adjusted analyses identified the independently predictive role of age in the higher EPO demand after 39 months.

EPO demand is directly calculated by Hb; thus, it may be related to baseline routine blood parameters, especially RBC and Hb variables. We found that the baseline Hb concentration was significantly higher in the lower follow-up EPO demand group. However, in the latter analyses, baseline Hb concentration showed no association with EPO demand. Neither was it an independent associated factor of higher EPO demand. Serum iron, ferritin, and PTH were also considered in the analyses; however, they were not associated with follow-up EDI. Although iron is one of the basic elements for the production of Hb, we did not find an association between iron and EPO demand (15).

We also attempted to identify the association between dialysis parameters (separated into predialysis and postdialysis). We revealed one novel finding that the predialysis potassium concentration was significantly higher in the higher EDI group. Further analyses also indicated that the predialysis potassium concentration was an independent associated factor/risk factor of higher EPO demand. Our present study identified a novel risk factor for follow-up EDI/EPO demand and predialysis potassium concentration. However, the mechanisms underlying this association have not been uncovered and may be associated with the renewal of erythrocytes and their energy demand (16). Others' studies have shown that potassium ion transport may play an important role in the production of erythrocytes as well as Hb (16–18). Furthermore, the associations have also been reported between potassium levels and Hb in the red cell units that undergo changes during storage and processing (19).

Anti-EPO antibodies have been reported in a small number of chronic renal disease patients who were administered EPO α (8). Others' studies have also found that anti-EPO antibody prevalence in ESRD patients who receive ESA may be associated with EPO resistance and demand, resulting in anemia (9, 20). Studies in patients with some autoimmune diseases and patients with HIV revealed high levels of anti-EPO antibodies (8, 21). Thus, EPO antibodies may participate in a variety of diseases. Importantly, we have identified that the baseline anti-EPO antibody level was associated with follow-up EPO demand. Previous studies suggest that EPO is associated with Hb loss (22). In addition, EPO antibodies have been demonstrated to mediate pure red cell aplasia after treatment with recombinant EPO products (22, 23). Furthermore, anemia treated with ESAs continuously failed to obtain a sustained response, which may also be caused by EPO antibodies. Finally, we identified that a higher baseline anti-EPO antibody level was an independent risk factor for higher EPO demand, which may provide a novel strategy for prevention or risk classification of anemia as well as the direction of ESA use.

## Limitations

We performed this prospective cohort study in 129 consecutive patients with ESRD who were undergoing dialysis and identified the predialysis potassium concentration, baseline anti-EPO antibody level, and age as independent risk factors for higher EPO demand. However, there are still three limitations to this study. First, the population size is relatively small. Second, there are many other factors that may be associated with EPO demand that should be included; however, we have not considered all of them owing to limited time and finances. Finally, the mechanisms underlying the associations between anti-EPO antibody and EPO demand have not been investigated, which warrants basic molecular research.

## Conclusion

The baseline anti-EPO antibody level combined with an older age and a higher predialysis potassium ion concentration are independent risk factors for a higher follow-up EPO demand in maintenance dialysis patients with ESRD, which also indicated that a lower baseline anti-EPO antibody level was an independent associated factor of lower EPO demand. The novel risk factors identified in this study may allow prevention or risk classification of anemia as well as guidance for ESA use.

## Data Availability Statement

The datasets generated for this study are available on request to the corresponding author.

## Ethics Statement

The studies involving human participants were reviewed and approved by the ethics committee of Xinqiao Hospital, Army Medical University (Third Military Medical University). The patients/participants provided their written informed consent to participate in this study.

## Author Contributions

YZ and JZ participated in the design of the study. YZ and S-ZB also drafted the manuscript and performed the statistical analysis. JZ reviewed and revised this manuscript critically for important intellectual content. YZ, ST, and KY collected the baseline history and baseline demographic data and the measurements of EPO and EPOR antibodies. The laboratory measurements were performed by YZ, YW, LN, and DW. The dialysis-related data were obtained by YW and WW.

### Conflict of Interest

The authors declare that the research was conducted in the absence of any commercial or financial relationships that could be construed as a potential conflict of interest.
