# Supplementary material for: Baseline Soluble Anti-erythropoietin Antibody Level Is an Independent Associated Factor for Follow-Up Erythropoietin Demand in Maintenance Dialysis Patients With End-Stage Renal Disease: A Prospective Cohort Study
Source: Front Med (Lausanne). 2020 Apr 7;7:109. doi: 10.3389/fmed.2020.00109 (PMC7154121; doi:10.3389/fmed.2020.00109)
Supplement: Supplementary file 1 [file Table_1.docx]

*Appendix*

Table S1 Correlation between follow-up EDI and other baseline parameters

| Variables | Follow-up EDI | |
| --- | --- | --- |
|  | Coefficient r | p value |
| *Demographics* |  |  |
| Age (years) | 0.287 | 0.001^**^ |
| BMI (kg/m^2^) | -0.19 | 0.797 |
| ***Baseline data in the initial of the study*** |  |  |
| *Routine blood examination parameters* |  |  |
| RBC (10^12^ /L) | -0.121 | 0.190 |
| Hb (g/dl) |  |  |
| Hct (L/L) | -0.065 | 0.482 |
| MCV (fl) | 0.164 | 0.073 |
| MCH (pg) | 0.122 | 0.184 |
| MCHC(g/dl) | -0.056 | 0.543 |
| RDW (%) | -0.086 | 0.543 |
| WBC (10^9^/L) | -0.018 | 0.849 |
| PCT (%) | -0.004 | 0.961 |
| PLT (10^9^/L) | -0.005 | 0.956 |
| MPV (fL) | -0.016 | 0.864 |
| PDW (%) | 0.052 | 0.576 |
| *Clinical data* |  |  |
| Serum iron (umol/L) | 0.028 | 0.759 |
| Ferritin (ug/L) | 0.256 | 0.005^**^ |
| PTH (pg/ml) | -0.049 | 0.592 |
| ***Dialysis parameters*** |  |  |
| Dialysis duration (month) | 0.201 | 0.028^*^ |
| Ultrafiltration volume (L) | -0.071 | 0.442 |
| *Pre-dialysis* |  |  |
| Urea (μmoI/L) | -0.074 | 0.420 |
| Cr (μmoI/L) | -0.226 | 0.013^*^ |
| Uric acid (μmol/L) | -0.098 | 0.286 |
| Calcium ion (mmol/L) | -0.073 | 0.428 |
| Serum phosphate (mmol/L) | -0.076 | 0.409 |
| Potassium ion (mmol/L) | 0.218 | 0.017^*^ |
| Serum sodium(mmol/L) | 0.004 | 0.964 |
| TCO_2_(mmol/L) | 0.061 | 0.551 |
| *Post-dialysis* |  |  |
| Urea (mmol/L) | -0.088 | 0.341 |
| Cr (umol/L) | -0.157 | 0.087 |
| Uric acid (umol/L) | 0.071 | 0.440 |
| Calcium ion(mmol/L) | -0.101 | 0.273 |
| Serum phosphate(mmol/L) | 0.060 | 0.513 |
| Potassium ion(mmol/L) | 0.136 | 0.139 |
| Serum sodium (mmol/L) | -0.117 | 0.205 |
| TCO_2_(umol/L) | 0.005 | 0.956 |
| Weight (kg) | -0.084 | 0.361 |
| KT/V | 0.042 | 0.652 |
| EPO and EPOR antibodies |  |  |
| EPO antibody OD | 0.000 | 0.999 |
| EPOR antibody OD | 0.274 | 0.002^**^ |

RBC: red blood cell;

Hct: hematocrit;

Hb: hemoglobin;

MCV: mean corpuscular volume;

MCH: mean corpuscular hemoglobin;

MCHC: mean corpuscular hemoglobin concentration;

RDW: red blood cell distribution width;

WBC: white blood cell;

PCT: plateletcrit;

PLT: platelet;

PDW: Platelet distribution width;

EPOA: EPO antibody;

EPOA+: EPO antibody positive;

EPORA: EPOR antibody;

EPORA+: EPOR antibody positive;

PTH: parathyroid hormone;

Cr: Creatinine;

EPO: erythropoietin mIU/ml;

OD: optical density;

KT/V:

*: p ≤ 0.05 between two groups;

**: p ≤ 0.01 between two groups.

Table S2 Univariate regressions for higher EDI (Q3 vs Q1)

| Variate | β | p | OR | 95%CI | |
| --- | --- | --- | --- | --- | --- |
|  |  |  |  | Lower border | Upper border |
| *Demographics* |  |  |  |  |  |
| Age (years) | 0.044 | 0.028^*^ | 1.045 | 1.005 | 1.086 |
| BMI (kg/m^2^) | 0.023 | 0.770 | 1.023 | 0.879 | 1.190 |
| ***Baseline data in the initial of the study*** | | | | | |
| *Routine blood examination parameters* | | | | | |
| RBC (10^12^ /L) | -0.483 | 0.248 | 0.617 | 0.271 | 1.401 |
| Hb (g/dl) | -0.017 | 0.320 | 0.983 | 0.952 | 1.016 |
| Hct (L/L) | -0.043 | 0.365 | 0.958 | 0.878 | 1.051 |
| MCV (fl) | 0.046 | 0.199 | 1.047 | 0.976 | 1.122 |
| MCH (pg) | 0.129 | 0.193 | 1.138 | 0.937 | 1.383 |
| MCHC(g/dl) | 0.018 | 0.328 | 1.019 | 0.982 | 1.057 |
| RDW (%) | -0.315 | 0.140 | 0.730 | 0.480 | 1.109 |
| WBC (10^9^/L) | -0.052 | 0.722 | 0.949 | 0.713 | 1.264 |
| PCT (%) | 0.111 | 0.979 | 1.118 | 0.001 | 6.710 |
| PLT (10^9^/L) | 0.000 | 0.901 | 1.000 | 0.993 | 1.008 |
| MPV (fL) | -0.071 | 0.718 | 0.932 | 0.635 | 1.368 |
| PDW (%) | 0.138 | 0.526 | 1.148 | 0.749 | 1.761 |
| *Clinical data* | | | | | |
| Serum iron (umol/L) | 0.029 | 0.449 | 1.029 | 0.955 | 1.109 |
| Ferritin (ug/L) | 0.001 | 0.176 | 1.001 | 1.000 | 1.002 |
| PTH (pg/ml) | 0.001 | 0.993 | 1.000 | 0.999 | 1.001 |
| ***Dialysis parameters*** | | | | | |
| Dialysis duration (month) | -0.017 | 0.320 | 0.983 | 0.952 | 1.016 |
| Ultrafiltration volume (L) | -0.144 | 0.442 | 0.866 | 0.600 | 1.250 |
| *Pre-dialysis* | | | | | |
| Urea (μmoI/L) | -0.049 | 0.319 | 0.952 | 0.865 | 1.048 |
| Cr (μmoI/L) | -0.003 | 0.042^*^ | 0.997 | 0.995 | 1.000 |
| Uric acid (μmol/L) | -0.006 | 0.082 | 0.994 | 0.988 | 1.001 |
| Calcium ion (mmol/L) | 0.220 | 0.842 | 1.246 | 0.143 | 10.886 |
| Serum phosphate (mmol/L) | -0.849 | 0.075 | 0.428 | 0.168 | 1.089 |
| Potassium ion (mmol/L) | 0.166 | 0.642 | 1.181 | 0.586 | 2.381 |
| Serum sodium(mmol/L) | 0.181 | 0.053 | 1.199 | 0.997 | 1.441 |
| TCO_2_(mmol/L) | 0.036 | 0.627 | 1.037 | 0.896 | 1.201 |
| *Post-dialysis* |  |  |  |  |  |
| Urea (mmol/L) | -0.107 | 0.290 | 0.899 | 0.737 | 1.095 |
| Cr (umol/L) | -0.002 | 0.369 | 0.998 | 0.994 | 1.002 |
| Uric acid (umol/L) | 0.001 | 0.957 | 1.000 | 0.998 | 1.012 |
| Calcium ion(mmol/L) | -1.265 | 0.368 | 0.282 | 0.018 | 4.421 |
| Serum phosphate(mmol/L) | -0.462 | 0.663 | 0.630 | 0.079 | 5.024 |
| Potassium ion(mmol/L) | 0.098 | 0.890 | 1.103 | 0.274 | 4.444 |
| Serum sodium (mmol/L) | 0.030 | 0.771 | 1.030 | 0.843 | 1.260 |
| CO_2_(umol/L) | 0.070 | 0.337 | 1.072 | 0.930 | 1.236 |
| Weight (kg) | -0.003 | 0.918 | 0.997 | 0.948 | 1.049 |
| KT/V | -0. 205 | 0.790 | 1.228 | 0.271 | 5.563 |
| EPO and EPOR antibodies |  |  |  |  |  |
| EPORA | -0.233 | 0.829 | 0.792 | 0.095 | 6.620 |
| EPOA | 1.414 | 0.007^**^ | 6.104 | 5.851 | 9.080 |

The regressions were performed using the Q1 interval as the reference, Q3 as the higher EDI.

*: p ≤ 0.05;

**: p ≤ 0.01.

Table S3 Adjusted regressions for higher EDI (Q3 vs Q1)

| Variables | β | p | OR | 95%CI | |
| --- | --- | --- | --- | --- | --- |
|  |  |  |  | Lower border | Upper border |
| Ferritin | 0.002 | 0.047^*^ | 1.002 | 1.000 | 1.003 |
| EPOA | 1.204 | 0.002^**^ | 5.898 | 1.894 | 6.751 |

The regressions were performed using the Q1 interval as the reference, Q3 as the higher EDI.

*: p ≤ 0.05;

**: p ≤ 0.01.

Table S4 Univariate regressions for higher EDI (Q4) vs. lower EDI (Q1-Q3)

| Variables | β | p | OR | | 95%CI | |
| --- | --- | --- | --- | --- | --- | --- |
|  |  |  |  |  | Lower border | Upper border |
| *Demographics* |  |  |  | |  |  |
| Age (years) | -1.054 | 0.574 | 0.348 | | 0.003 | 1.074 |
| BMI (kg/m^2^) | -2.596 | 0.375 | 0.074 | | 0.032 | 0.255 |
| ***Baseline data in the initial of the study*** | | | | | | |
| *Blood routine examination parameters* | | | | | | |
| RBC (10^12^ /L) | -2.129 | 0.378 | 0.119 | 0.033 | | 0.412 |
| Hb (g/dl) | -22.102 | 0.995 | 0.000 | 0.000 | | 0.001 |
| Hct (L/L) | -24.124 | 0.990 | 0.000 | 0.000 | | 0.001 |
| MCV (fl) | 18.594 | 0.992 | >1000 | 0.981 | | >10000 |
| MCH (pg) | -1.011 | 0.588 | 0.364 | 0.091 | | 0.826 |
| MCHC(g/dl) | -0.745 | 0.682 | 0.475 | 0.133 | | 1.023 |
| RDW (%) | 18.63 | 0.996 | >1000 | 0.686 | | >10000 |
| WBC (10^9^/L) | -38.525 | 0.905 | 0.000 | 0.000 | | 0.001 |
| PCT (%) | -1.506 | 0.461 | 0.222 | 0.018 | | 0.567 |
| PLT (10^9^/L) | -0.649 | 0.772 | 0.523 | 0.091 | | 1.003 |
| MPV (fL) | -22.112 | 0.995 | 0.000 | 0.000 | | 0.001 |
| PDW (%) | -21.401 | 0.995 | 0.000 | 0.000 | | 0.001 |
| *Clinical data* |  |  |  |  | |  |
| Serum iron (umol/L) | -0.936 | 0.613 | 0.392 | 0.102 | | 1.023 |
| Ferritin (ug/L) | 7.295 | <0.001** | 1472.917 | 1.973 | | 2237.189 |
| PTH (pg/ml) | -10.517 | <0.001^**^ | 0.000 | 0.000 | | 0.329 |
| *Dialysis parameters* |  |  |  |  | |  |
| Dialysis duration (month) | -2.668 | 0.151 | 0.069 | 0.035 | | 1.218 |
| Ultrafiltration volume (L) | -2.136 | 0.242 | 0.118 | 0.097 | | 0.851 |
| *Pre-dialysis* |  |  |  |  | |  |
| Urea (μmoI/L) | -17.005 | 0.887 | 0.000 | 0.000 | | 0.002 |
| Cr (μmoI/L) | -10.210 | 0.020^*^ | 0.000 | 0.000 | | 0.007 |
| Uric acid (μmol/L) | -7.241 | 0.018^*^ | 0.000 | 0.000 | | 0.015 |
| Calcium ion (mmol/L) | -22.189 | 0.993 | 0.000 | 0.000 | | 0.001 |
| Serum phosphate (mmol/L) | 18.583 | 0.992 | >1000 | 0.768 | | >10000 |
| Potassium ion (mmol/L) | 18.601 | 0.992 | >1000 | 0.931 | | >10000 |
| Serum sodium(mmol/L) | -0.889 | 0.629 | 0.411 | 0.301 | | 0.827 |
| TCO_2_(mmol/L) | -1.092 | 0.563 | 0.336 | 0.287 | | 1.001 |
| *Post-dialysis* |  |  |  |  | |  |
| Urea (mmol/L) | -10.052 | 0.020^*^ | 0.000 | 0.000 | | 0.008 |
| Cr (umol/L) | -8.886 | 0.003^**^ | 0.000 | 0.000 | | 0.014 |
| Uric acid (umol/L) | -1.889 | 0.406 | 0.151 | 0.012 | | 1.032 |
| Calcium ion(mmol/L) | -22.233 | 0.994 | 0.000 | 0.000 | | 0.001 |
| Serum phosphate(mmol/L) | -1.028 | 0.582 | 0.358 | 0.087 | | 1.093 |
| Potassium ion(mmol/L) | -3.379 | 0.079 | 0.034 | 0.007 | | 0.982 |
| Serum sodium (mmol/L) | -22.307 | 0.994 | 0.000 | 0.000 | | 0.001 |
| TCO_2_(umol/L) | -22.623 | 0.992 | 0.000 | 0.000 | | 0.001 |
| KT/V | -7.381 | 0.786 | 0.001 | 0.000 | | 0.002 |
| EPO and EPOR antibodies |  |  |  |  | |  |
| EPORA OD | -23.928 | 0.590 | 0.000 | 0.000 | | <0.001 |
| EPOA OD | 3.434 | 0.041^*^ | 31.00 | 1.187 | | 40.238 |

The regressions were performed using ordinal logistic regression analyses, we have just listed the Q4 EDI vs other intervals (Q1-3) due to too many data.

*: p ≤ 0.05;

**: p ≤ 0.01.

Table S5 Adjusted regressions

| Variables | β | p | OR | 95%CI | |
| --- | --- | --- | --- | --- | --- |
|  |  |  |  | Lower border | Upper border |
| Ferritin (ug/L) | 1.909 | 0.044^*^ | 6.746 | 1.028 | 9.976 |
| PTH (pg/ml) | -2.753 | 0.035^*^ | 0.064 | 0.009 | 0.148 |
| EPOA OD | 1.673 | 0.045^*^ | 5.328 | 1.072 | 8.735 |

The regressions were performed using ordinal logistic regression analyses, we have just listed the Q4 EDI vs other intervals (Q1-3) due to too many data.

*: p ≤ 0.05.
